# Proteomics Analysis of Myocardial Tissues in a Mouse Model of Coronary Microembolization

**DOI:** 10.3389/fphys.2018.01318

**Published:** 2018-09-19

**Authors:** Ao Chen, Zhangwei Chen, Yan Xia, Danbo Lu, Jianguo Jia, Kai Hu, Aijun Sun, Yunzeng Zou, Juying Qian, Junbo Ge

**Affiliations:** Department of Cardiology, Shanghai Institute of Cardiovascular Diseases, Zhongshan Hospital, Fudan University, Shanghai, China

**Keywords:** coronary microembolization, proteomics, bioinformatics, metabolism, cytoskeleton

## Abstract

Coronary microembolization (CME) is an important clinical problem, and it is related to poor outcome. The specific molecular mechanisms of CME are not fully understood. In the present study, we established a mice model of CME. Isobaric tags for relative and absolute quantitation (iTRAQ) and liquid chromatography coupled with tandem mass spectrometry (LC-MS/MS) technologies identified 249 differentially expressed proteins in the myocardial tissues of CME mice as compared with sham-operated mice. Bioinformatics analysis demonstrated that these differentially expressed proteins were enriched in several energy metabolism or cytoskeleton organization related processes or pathways. Quantitative PCR and Western blotting validation experiments revealed that succinate dehydrogenase (SDHA and SDHB) were upregulated, Rho GDP dissociation inhibitor α (RhoGDIα) and Filamin-A (FLNA) were downregulated significantly in CME mice. These findings indicated that the alternations of the cytoskeleton and energy metabolism pathways play important roles in the pathogenesis of CME, future studies are warranted to verify if targeting these molecules might be useful to alleviate CME injury or not.

## Introduction

Coronary microembolization (CME) is a critical and frequent clinical complication, usually induced by spontaneous rupture of atherosclerotic plaque in acute coronary syndrome or iatrogenic rupture during percutaneous coronary intervention (Heusch et al., 2004, 2009; Skyschally et al., 2006). CME captures increasing attention due to the undesirable consequences, such as regional myocardial contractile dysfunction and malignant arrhythmias (Erbel and Heusch, 2000). CME patients are also associated with poor clinical outcomes (Henriques et al., 2002; Fokkema et al., 2009).

It is known that several pathophysiological processes are involved in CME. Leukocyte infiltration is one of the core features in the occurrence of microinfarcts caused by CME, so inflammatory response might be responsible for the contractile dysfunction following CME (Dorge et al., 2000). It is worth mentioning that tumor necrosis factor-α (TNF-α) could also play a causal role in CME-related contractile dysfunction (Heusch et al., 2014), and most likely through triggering cardiomyocyte apoptosis and sphingosine production (Thielmann et al., 2002; Chen et al., 2014). Moreover, the oxidation of contractile proteins may contribute to the contractile impairment following CME (Canton et al., 2006). Till now, the specific molecular mechanisms related to CME remain largely elusive. Further understanding of the underlying mechanisms of CME might help to develop novel therapeutic strategies to alleviate the consequences of CME and improve outcomes of CME patients.

Modern approaches and methodologies of system biology analysis are becoming increasingly important in clarifying disease mechanisms. Novel proteomics techniques, liquid chromatography coupled with tandem mass spectrometry (LC-MS/MS) technology are powerful tools to detect protein changes and low-abundance protein alternations in injured tissue samples and experimental disease models. Isobaric tags for relative and absolute quantitation (iTRAQ), a MS-based proteomics approach, is helpful to compare protein expression levels between multiple samples, and can provide accurate mass measurement of critical changes in the amounts of proteins (Unwin et al., 2010). Using proteomic methods, previous studies have uncovered several novel pathophysiological processes, potential therapeutic targets, or biomarkers in the context of myocardial infarction, cardiac remodeling, and dilated cardiomyopathy (Liu et al., 2017; Yang et al., 2017; Jacob et al., 2018; Turkieh et al., 2018). However, there is only scanty in-depth analysis of the protein changes following CME.

In the present study, iTRAQ labeling combined with LC-MS/MS techniques were utilized to investigate the differentially expressed proteins and pathways in the cardiac tissues of CME mice.

## Materials and Methods

### Animals

Adult male C57BL/6 mice (8 weeks old, 22–25 g) were purchased from LingChang BioTech Co. Ltd. (Shanghai, China). Mice were randomly divided into the following two groups: sham-operation group (SO, *n* = 12) and CME group (CME, *n* = 12). The mouse CME model was established as described in our previous study (Cao et al., 2016). Briefly, mechanically ventilated mice were anesthetized with 1.5% isoflurane (Baxter International Inc., United States), and then thoracotomy was performed to expose the ascending aorta and heart. Simultaneous with the occlusion of the ascending aorta for 15 s, a total of 500,000 polyethylene microspheres (Dyno Particles AS, Norway) with an average diameter of 9 μm were injected into the left ventricle chamber. The SO group was injected with saline instead of microspheres. Then, after the measurement of cardiac function, all mice were sacrificed, and the hearts were quickly sampled and stored in liquid nitrogen for further analysis.

The investigation was approved by the Animal Care and Use Committee of Fudan University Zhongshan Hospital, and all protocols conformed to the Guide for the Care and Use of Laboratory Animals published by the US National Institutes of Health (NIH Publication No. 85-23, revised 1996).

### Detection of Cardiac Function

Three days after polyethylene microspheres or saline injection, transthoracic echocardiography was performed using Vevo770 ultrasound systems (VisualSonics, Canada) as previously described (Xia et al., 2017). The mice were anesthetized with 2% isoflurane (Baxter, Denmark) and laid supine on a heated platform. Left ventricular ejection fraction (LVEF), left ventricular end-diastolic diameter (LVEDD), left ventricular end-systolic diameter (LVESD), and fractional shortening (FS) were measured and calculated from M-mode images.

### Histological Analysis

Tissues from the apical heart region were dissected, fixed in 4% paraformaldehyde, embedded in paraffin, and then cut into 5 μm sections. For morphological observations, the sections were stained with hematoxylin–eosin (HE) or Masson trichrome to detect cardiac morphology and collagen deposition, respectively. For immunohistochemical analyses, the paraffin sections were deparaffinized in xylene, rehydrated in ethanol, and soaked in 3% hydrogen peroxide to quench endogenous peroxidase activity. The sections were incubated with anti-F4/80 (Abcam, UK) at 4°C overnight. They were then rinsed in PBS and incubated with secondary antibody conjugated to HRP followed by a diaminobenzidine (DAB) substrate and counterstaining with hematoxylin. Histological images were obtained using an Olympus BX-51 light microscope (Olympus America Inc., United States) and measured using Image J software (Version 1.50, National Institutes of Health, United States).

### Protein Preparation

Two heart samples from each group were randomly selected for proteomics analysis. Each sample (50 mg) was homogenized in lysis buffer containing 1% protease inhibitor cocktail (Thermo Fisher Scientific, United States), and then ultra-sonicated to extract total proteins. The suspension was centrifuged at 14,000 *g* for 45 min at 4°C. Next, the supernatant was collected, and protein concentration was determined by bicinchoninic acid (BCA) assay method. Protein (200 μg) was reduced with 100 mM dithiothreitol (DTT) at 100°C for 5 min and subsequently alkylated with 50 mM iodoacetamide (IAA) for 30 min in darkness. All samples were digested with trypsin (Promega, United States) at 37°C for 16–18 h, reconstituted using 40 μl dissolution buffer, and then labeled with tags with an iTRAQ reagent kit (AB Sciex, United States) following the manufacturer’s protocol.

### LC-MS/MS Analysis

Labeled samples were separated through online reversed-phase chromatography using Easy nLC1000 system (Thermo Fisher Scientific, United States). The peptides were autoloaded into a C18 trap column (2 cm × 100 μm, 5 μm; Thermo Fisher Scientific, United States), and subsequently eluted into a C18 analytical column (75 μm × 100 mm, 3 μm; Thermo Fisher Scientific, United States) for gradient elution at a flow rate of 250 nL/min for 120 min. LC-MS/MS was conducted using a Q-Exactive (Thermo Fisher Scientific, United States) mass spectrometer. The procedure was performed in positive ion mode with MS1 survey scan (m/z: 300–1800) at a resolution of 70,000, followed by 10 higher-energy collisional dissociation (HCD) type MS2 scans with a resolution of 17,500.

### Protein Identification

The MS raw files were processed using Proteome Discoverer 1.3 software (Thermo Fisher Scientific, United States). Database searching of the raw data was conducted with Mascot 2.2 against the UniProt mouse database which included 17,197 protein sequences. The following parameters were used in the Mascot search: two missed trypsin cleavage sites, carbamidomethyl fixed modification, methionine oxidation variable modifications, ±20 ppm peptide mass tolerance, 0.1 Da fragment mass tolerance and decoy database pattern. Peptides were identified and filtered to 1% false discovery rate (FDR).

### Bioinformatics Analysis

Proteins were regarded as differentially expressed if they revealed more than 1.2-fold change (≥1.2 or ≤0.83) and the *P*-value was <0.05 based on Student’s *t*-test between the groups. Matrix2png program^1^ was used for making heatmap visualizations of differentially expressed proteins. The analysis of Gene Ontology (GO) enrichment, Kyoto Encyclopedia of Genes and Genomes (KEGG) pathways, and protein–protein interaction (PPI) network was preceded using the online bioinformatics data analysis tool OmicsBean^2^.

### Quantitative PCR

Total RNA was extracted from heart samples using TRIzol Reagent (Invitrogen Biotechnology, United States). cDNA was synthesized from 400 ng of total RNA using PrimeScript RT Master Mix (Takara Biomedical Technology, Japan). Quantitative PCR (qPCR) was performed using SYBR Premix Ex Taq (Takara Biomedical Technology, Japan) on a CFX96 Real-Time PCR Detection System (Bio-Rad Laboratories, United States) following the manufacturer’s protocol. Succinate dehydrogenase (Sdha and Sdhb), Rho GDP dissociation inhibitor α (Arhgdia), and Filamin-A (Flna) were selected as target genes. Glyceraldehyde-3-phosphate dehydrogenase (Gapdh) expression was used as internal reference, and the relative level of target gene expression was calculated by the 2^-ΔΔCq^ method. The sequences of the specific primers for each gene were listed in **Table 1**.

**Table 1:** Primer sequences for quantitative PCR.

Target Gene	Gene ID	Forward Sequence (5′ → 3′)	Reverse Sequence (5′ → 3′)
Sdha	66945	GGAACACTCCAAAAA CAGACCT	CCACCACTGGGTAT TGAGTAGAA
Sdhb	67680	AATTTGCCATTTACCG ATGGGA	AGCATCCAACACCA TAGGTCC
Arhgdia	192662	AAGGACGATGAAA GCCTCCG	GGTCAGTCGAGTC ACAATGACA
Flna	192176	GAGTTCGGCATTT GGACTAGG	GGGCTATCAGGTAT GTGCTCC
Gapdh	14433	CGTGCCGCCTGGA GAAACC	TGGAAGAGTGGGAG TTGCTGTTG


### Western Blotting

Total protein (40 μg) was loaded onto a 8–12% sodium dodecyl sulfate–polyacrylamide gel, and then transferred to polyvinylidene fluoride (PVDF) membranes (Millipore, United States). The membranes were blocked in Tris-buffered saline +0.1% Tween-20 (TBST) containing 5% bovine serum albumin (BSA) for 1 h and then incubated with primary antibody against SDHA, SDHB, RhoGDIα, FLNA (Abcam, United Kingdom) and GAPDH (Cell Signaling Technology, United States) at 4°C overnight. GAPDH expression was used as internal control. The membranes were incubated with secondary antibody (Cell Signaling Technology, United States) for 1.5 h after three washes. The antigen–antibody complexes were detected using an enhanced chemiluminescence kit (Thermo Fisher Scientific, United States).

### Statistical Analysis

Continuous variables were analyzed for distribution status. Data with normal distribution were presented as mean ± SEM. Differences between groups were determined by Student’s *t*-test. *P* values <0.05 were considered statistically significant. All data analyses were performed using GraphPad Prism version 5.0 software (GraphPad Prism, United States).

## Results

### CME-Induced Cardiac Contractile Dysfunction

**FIGURE 1:**
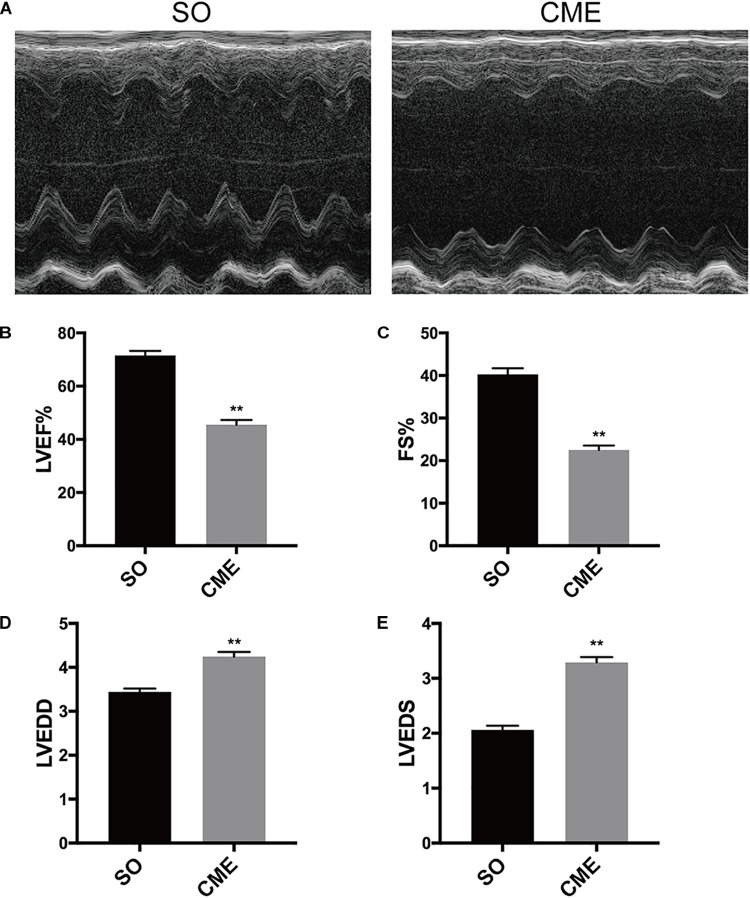
Cardiac function detection by echocardiography. **(A)** Representative M-mode echocardiograms. Group data for **(B)** left ventricular ejection fraction (LVEF), **(C)** fractional shortening (FS), **(D)** left ventricular end-diastolic diameter (LVEDD), and **(E)** left ventricular end-systolic diameter (LVEDS). ^∗∗^*P* < 0.01; *n* = 12 per group.

Echocardiography results demonstrated that cardiac systolic function was impaired as characterized by significant decreased LVEF and FS in the CME group as compared with the respective values in SO group (*P* < 0.01) (**Figures 1A–C**). In addition, LVEDD and LVEDS were significantly increased in CME mice (*P* < 0.01) (**Figures 1D,E**).

### CME-Induced Cardiac Morphological Changes

**FIGURE 2:**
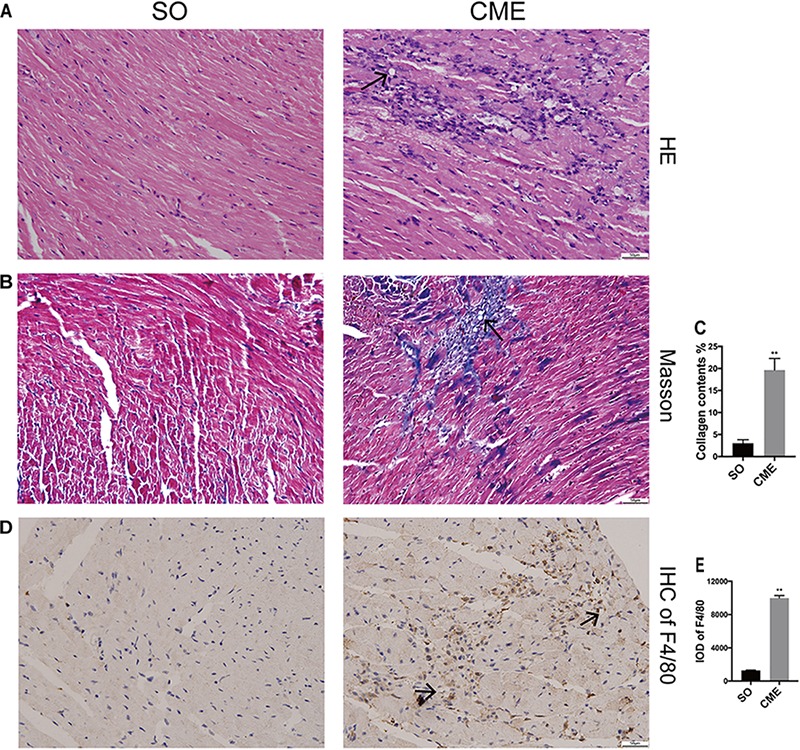
Histological analysis of the hearts from three groups. **(A)** Representative HE-staining micrographs to visualize myocytes arrangement. **(B)** Cardiac sections were stained with Masson to visualize collagen deposition around the microspheres. **(C)** The quantitative analysis of collagen contents (%). **(D)** Immunohistochemical staining of F4/80 to reflect macrophage infiltration. **(E)** Integrated optical density (IOD) of F4/80 in cardiac tissues. *n* = 6 per group. Scale bars = 50 μm. Black arrows indicated the microspheres. ^∗∗^*P* < 0.01.

HE staining revealed disorganized myocytes around the microspheres deposited in coronary microcirculation of hearts in the CME group (**Figure 2A**). Notably, hearts of CME mice exhibited considerably higher amounts of collagen, especially around the microspheres, as compared with hearts from the SO group (**Figures 2B,C**). CME also resulted in enhanced inflammatory cells infiltrating in the myocardium, primarily localizing around the microspheres, as demonstrated by the accumulation of enriched F4/80^+^ macrophages in the CME hearts (**Figures 2D,E**).

### Proteomics Analysis

**FIGURE 3:**
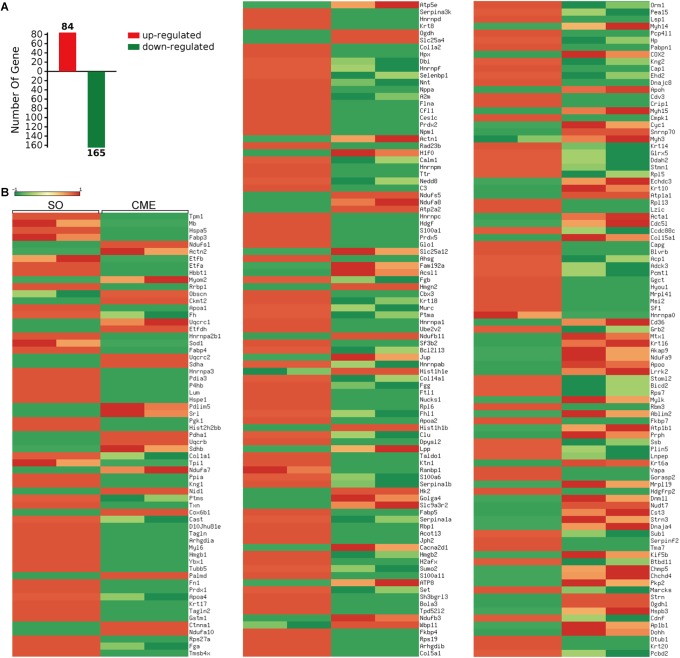
Proteomics analysis of the differentially expressed proteins. **(A)** Numbers of upregulated and downregulated proteins in CME mice. **(B)** Heatmap of the expression levels of the 249 differentially expressed proteins, red and green reflected high and low expression levels, respectively.

A total of 1145 mouse proteins were unambiguously identified in this proteomics analysis. Comparison between the two groups revealed that 84 proteins were upregulated and 165 downregulated in the CME group compared with the SO group (**Figure 3A**). Then, by using heatmap, the expression levels of these 249 differentially expressed proteins were elaborately and visually displayed (**Figure 3B**).

### GO Enrichment and Pathway Analysis

**FIGURE 4:**
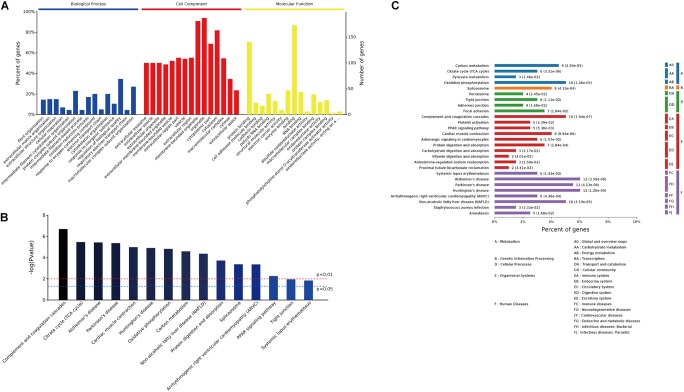
GO enrichment and pathway analysis of the 249 differentially expressed proteins. **(A)** Bar chart depicting the GO classification of the differentially expressed proteins in terms of biological process, cellular component, and molecular function. **(B)** Top 15 KEGG pathways enriched by the differentially expressed proteins. **(C)** Classification of the enriched KEGG pathways.

GO enrichment analysis was conducted on the 249 differentially expressed proteins to further explore their biological functions. The top 15 significantly enriched GO terms were depicted in **Figure 4A**. In biological process analysis, fibril organization was the most striking term, followed by other cytoskeleton or metabolism related processes, for instance, cytoskeleton organization, cellular respiration, oxidation–reduction process, response to oxygen-containing compound, and electron transport chain. Concerning cell component, most of these annotated proteins were localized in organelles or membrane-bounded organelles. Molecular function analysis revealed that protein binding (cell adhesion molecule binding, cadherin binding, and protein complex binding) and metabolic enzymes activity (electron carrier activity, disulfide oxidoreductase activity, and phosphatidylcholine-sterol O-acyltransferase activator activity) were the predominant terms (**Figure 4A**). Top 15 KEGG pathways were listed in **Figure 4B**, metabolism pathways, including citrate cycle, oxidative phosphorylation, and carbon metabolism, were significantly enriched (**Figures 4B,C**). In addition, peroxisome proliferator-activated receptor (PPAR) signaling pathway, which transcriptionally regulates myocardial energy metabolism (Finck, 2007), was found to be tangled following CME.

### PPI Analysis

**FIGURE 5:**
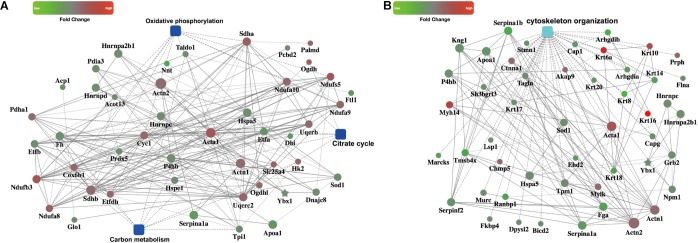
PPI networks. **(A)** PPI analysis of the metabolism related pathways, including carbon metabolism, oxidative phosphorylation, and citrate cycle **(B)** PPI analysis of the cytoskeleton organization process.

We then focused on metabolism pathways and cytoskeleton organization changes based on the enriched GO terms or pathways. PPI analysis using OmicsBean online tool provided us distinct networks formed by interacting proteins with visual displayed data to get insight into the pathogenesis involved in CME. Three metabolism pathways (carbon metabolism, oxidative phosphorylation, and citrate cycle) refined by KEGG metabolism classification, were enrolled for PPI analysis (**Figure 5A**). Cytoskeleton organization process related proteins were shown in **Figure 5B**.

### Quantitative PCR and Western Blotting Validation

**FIGURE 6:**
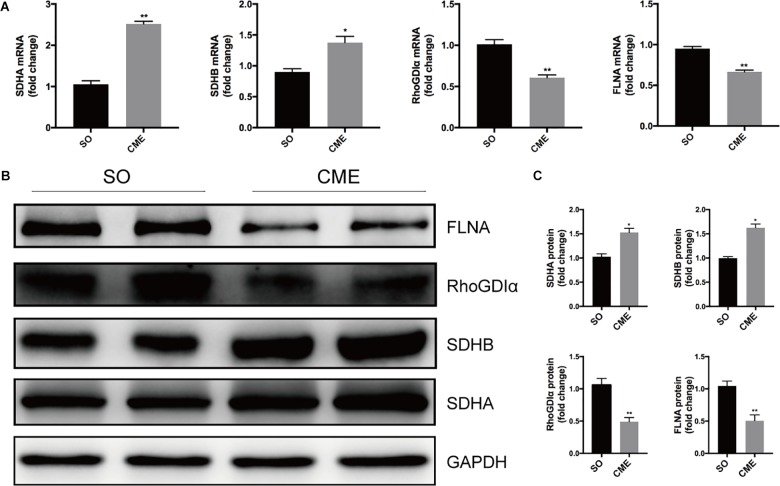
Validation of the proteomics results by qPCR and Western blotting. **(A)** Results of qPCR for the mRNA levels of SDHA, SDHB, RhoGDIα, and FLNA. **(B)** Results of Western blotting for SDHA, SDHB, RhoGDIα, and FLNA. **(C)** Densitometric analysis of relative protein expressions, GAPDH was used as internal control. *n* = 4 per group. ^∗^*P* < 0.05, ^∗∗^*P* < 0.01.

To verify the proteomics results and to validate the altered myocardial energy metabolism and cytoskeleton organization following CME, we first limited the target proteins in energy metabolism and cytoskeleton organization processes (PPI analysis in **Figure 5**). To further narrow the scope of the target proteins, we scanned literatures about the proteins listed in the PPI analysis, and selected the proteins which have a direct and particularly close connection to energy metabolism and cytoskeleton organization for validation. Ultimately, two enzymes involved in the citrate cycle, SDHA and SDHB, and two proteins participated in cytoskeleton organization, RhoGDIα and FLNA, were enrolled for qPCR and Western blotting analysis. In the transcriptional level, we observed a remarkable upregulation of SDHA and SDHB, and downregulation of RhoGDIα and FLNA mRNA expression in the CME group (**Figure 6A**). The protein expression levels of SDHA and SDHB were also significantly increased, RhoGDIα and FLNA decreased in the CME group as compared with the SO group (**Figures 6B,C**), which were all consistent with the proteomics results.

## Discussion

Cardiac contractile dysfunction, myocardial fibrosis, and enhanced inflammation were evidenced in this mice model of CME. To further understand the pathophysiological processes of CME, the differential protein expression pattern was displayed between the sham-operated and CME mice using iTRAQ and LC-MS/MS based proteomics technology, and then verified by qPCR and Western blotting analysis. A total of 249 proteins were revealed to be differentially expressed following CME. GO enrichment and KEGG pathway analysis suggested that several cytoskeleton or metabolism related processes, such as fibril organization, carbon metabolism, and citrate cycle, were perturbed in CME mice. The extensive proteomics analysis allowed for the first time an in-depth observation of the protein alterations in CME.

It is well known that heart exhibits a high capacity of adenosine 5′-triphosphate (ATP) production, which is indispensable for continuous mechanical contraction (Suga, 1990). Perturbations in ATP production resulting from energy metabolism disturbances might impair the cardiac contractile function. The term “metabolic remodeling” in cardiac hypertrophy and heart failure has been well established and may provide certain guidance for new therapeutic directions (Peterzan et al., 2017; Bertero and Maack, 2018). It is noteworthy that previous studies on CME have largely focused on myocardial apoptosis or cardiac inflammation (Dorge et al., 2002; Skyschally et al., 2004; Chen et al., 2014), while energy metabolism disturbances in case of CME have received little deliberate attention. Present proteomics analysis depicted several energy metabolic abnormalities in CME mice, especially citrate cycle, a key pathway involved in the oxidation of glucose, fatty acids, and amino acid metabolism, was found to be dysregulated. Besides, the metabolism upstream modulator, PPAR signaling pathway, was also enriched as detected by the KEGG pathway analysis in CME mice. PPAR-targeted drugs have been demonstrated to confer cardioprotection in cardiovascular diseases (Shiomi et al., 2002; Bulhak et al., 2006), it is therefore possible that these drugs might also serve as a feasible strategy in the treatment of CME. Meanwhile, KEGG pathway analysis implied cardiac muscle contraction impairment in CME mice, indicating that the cardiac dysfunction following CME may be at least partly explained by the impaired ATP production due to energy metabolic abnormalities.

In addition, PPI analysis revealed that two subunits of succinate dehydrogenase, SDHA and SDHB, were the altered proteins, which were known to be involved in the citrate cycle. Succinate is not only a metabolic intermediate during citrate cycle but also involves in the mitochondrial reactive oxygen species (ROS) production process via reverse electron transport (RET) and thereby enhances the inflammatory responses (Tannahill et al., 2013; Chouchani et al., 2014). Previous study has shown that SDH is responsible for the accumulation and oxidation of succinate during cardiac ischemia/reperfusion (I/R). Inhibiting SDH could reduce ROS production and protect against I/R injury (Chouchani et al., 2014). Heatmap analysis, qPCR and Western blotting analysis revealed an increased expression level of SDHA and SDHB following CME in the present study, which might be a source of ROS production in CME mice. Not surprisingly, KEGG pathway analysis also demonstrated abnormal oxidative phosphorylation in CME mice, in line with the alteration of oxidation–reduction biological process by GO analysis. Previous studies showed that ROS accumulation could lead to oxidative modification of tropomyosin (Canton et al., 2006). Moreover, ROS generation may amplify inflammatory responses via distinct molecular pathways (Naik and Dixit, 2011), both redox signaling and inflammation could enhance cardiac fibrosis by inducing the proliferation and differentiation of fibroblasts and the deposition of collagen (Seddon et al., 2007). In line with the proteomics results, pathological analysis demonstrated increased inflammation and collagen deposition in this CME mice model. It is to note that above changes hinting mitochondrial dysfunction were also the common disease features of various neurodegenerative disorders including Alzheimer’s, Parkinson’s, and Huntington’s diseases (Taylor et al., 2003; Liu et al., 2014; Schapira et al., 2014). Thus, our results also indicated the close association of cardiac and neuroembolism-related diseases, showing ischemic cardiac and neurological diseases share the common pathogenesis including enhanced oxidative stress and mitochondrial dysfunction.

The proteomics analysis used in the present study revealed Arhgdia-encoded protein RhoGDIα was decreased in CME mice, this finding was further confirmed by Western blotting and qPCR analysis. It is known that the ubiquitously expressed protein RhoGDIα interacts with several Rho GTPases and maintains them in an inactive state by inhibiting the dissociation of GDP from them (Boulter et al., 2010). Rho family proteins could regulate several pathways including actin organization, apoptosis, proliferation, migration, and transformation. RhoGDIα thus serves as a pleiotropic modulator (Cho et al., 2012; Okina et al., 2012). Previous study reported that RhoGDIα might exert influence on cardiac electrical activity via regulating expression and/or activity of cardiac structural proteins, such as connexin 40 (Loirand et al., 2013), and this might be linked with the pathogenesis of various malignant arrhythmias following CME (Frink et al., 1988). Another cytoskeleton-related protein FLNA was also found to be downregulated following CME in the present study. Previous studies demonstrating that FLNA was essential for actin cytoskeleton remodeling and intercellular junctions during heart development (Feng et al., 2006; Metais et al., 2018), our finding hinted a possible role of FLNA in post-CME remodeling.

Admittedly, some limitations of this study need to be acknowledged. First, quantitative proteomics alone is not enough to unearth the metabolic changes comprehensively, because it can only discover the expression changes of metabolic enzymes in protein level. However, the alterations of enzyme activities are also important for metabolism. Thus, proteomics along with kinomics or metabolomics are necessary for exploring the more detailed metabolic alterations following CME. Second, all the analyses were made on tissue samples obtained at 3 days after CME, future studies are warranted to clarify the long-term metabolic changes or remodeling-induced changes in this CME model. Third, although we have validated the expression changes of four proteins in this study, further functional experiments are warranted to evaluate the detailed roles of these differentially expressed proteins. Last but not least, although the mice model is a powerful tool to uncover pathogenesis of human diseases, the differences in gene expression patterns between them should be taken into account, further investigations in human cardiac tissues with coronary microembolism are needed to clarify the disease pathogenesis.

## Conclusion

Using a mice model of CME and state-of-the-art iTRAQ labeling and LC-MS/MS technology, we provide the first proteomic characterization of myocardial tissues of CME mice. Bioinformatics analysis indicates significant alternations of the cytoskeleton and metabolism-related proteins following CME, future studies are warranted to verify if strategies targeting these differentially expressed molecular proteins and signaling pathways might confer novel therapeutic options for CME or not.

## Data Availability Statement

The datasets for this study will be available from the corresponding authors on reasonable request.

## Author Contributions

JQ and JG designed the study and obtained the funding. AC and ZC carried out the animal models, performed the proteomics analysis and validation, collected and analyzed the data, and drafted the manuscript. YX and DL assisted in performing qPCR and Western blotting. JJ assisted in establishing the CME models. KH, AS, and YZ assisted in compiling data and editing the manuscript. All authors have reviewed the manuscript.

## Conflict of Interest Statement

The authors declare that the research was conducted in the absence of any commercial or financial relationships that could be construed as a potential conflict of interest.

## Footnotes

1 http://chibi.ubc.ca/matrix2png/bin/matrix2png.cgi

2 http://www.omicsbean.com:88
